# An improved system to generate recombinant canine distemper virus

**DOI:** 10.1186/s12917-023-03830-x

**Published:** 2024-04-27

**Authors:** Huai Cheng, Hewei Zhang, Huayun Zhang, Huanchang Cai, Min Liu, Mingen Yu, Meihua Xiang, Shubo Wen, Jingqiang Ren

**Affiliations:** 1 Wenzhou Key Laboratory for Virology and Immunology, Institute of Virology, Wenzhou University, Wenzhou, China; 2 College of Food and Drugs, Luoyang Polytechnic, Luo Yang, China; 3 Animal Diseases and Public Health Engineering Research Center of Henan Province, Luoyang, China; 4 Research and Development Department, Hangzhou Goodhere Biotechnology Co., Ltd, Hangzhou, China; 5 Preventive Veterinary Laboratory, College of Animal Science and Technology, Inner Mongolia Minzu University, Tongliao, China

**Keywords:** Canine distemper virus, Negative-sense RNA, Reverse genetic system, Recombinant virus, Minireplicon

## Abstract

**Background:**

Canine distemper virus (CDV) is a pathogen with the capability of cross-species transmission. It has crossed the species barrier to infect many other species, and its host range is expanding. The reverse genetic platform, a useful tool for scientific research, allows the generation of recombinant viruses from genomic cDNA clones in vitro.

**Methods:**

To improve the reverse genetic system of CDV, a plasmid containing three independent expression cassettes was constructed for co-expression of the N, P, and L genes and then transfected with a full-length cDNA clone of CDV into Vero cells.

**Results:**

The results indicated that the established rescue system has the advantages of being more convenient, easy to control the transfection ratio, and high rescue efficiency compared with the conventional reverse genetics system.

**Conclusion:**

This method not only reduces the number of transfection plasmids, but also improves the rescue efficiency of CDV, which could provide a reference for the recovery of other morbilliviruses.

## Background

Canine distemper virus (CDV) is the etiologic agent of canine distemper (CD), an acute and highly contagious infectious disease that occurs in a variety of animals, including canines, ferrets, tigers, lions, raccoons, pandas, nonhuman primates, and other animal species [1–6]. CDV is an enveloped, non-segmented, single-stranded negative-sense RNA virus with a genome of approximately 16 kb consisting of six genes that encode the nucleocapsid protein (N), phosphoprotein (P), matrix protein (M), fusion protein (F), hemagglutinin protein (H), and large protein (L) [7, 8].

Structurally, viral RNA genomes are packaged by N proteins into helical ribonucleoprotein complexes (RNPs), which are subsequently recognized as templates for mRNA synthesis and replication by viral RNA-dependent RNA polymerase (RdRP). The core domains of the RdRP complex are the viral P and L proteins, mediating the attachment of N to genomic RNA and providing enzymatic activities needed for mRNA synthesis, respectively [9, 10]. Therefore, naked viral mRNA alone is not infectious, and transfection of negative-stranded RNA into cells does not produce viral particles. To be infectious, providing RNP to host cells during viral transcription is necessary.

The conventional reverse genetic systems for CDV rescue involve four or five plasmids, one of which is the full-length cDNA clone of the CDV genome. The others are helper plasmids that encode three (N, P, and L) or four (N, P, L, and T7 RNA polymerase) proteins essential for transcription and replication, as well as in some cases requiring the involvement of T7-expressing helper viruses [11–14]. In the present study, to reduce the number of co-transfected plasmids and address contamination problems from helper viruses, we established a two-plasmid reverse genetics system for recovering CDV from cloned cDNA, which will be useful in CDV virological research.

## Methods

### Virus and cells

African green monkey kidney (Vero) cells and BSR cells (a clone of BHK-21) were cultured in Dulbecco’s modified Eagle’s medium (DMEM; Thermo Fisher Scientific, Shanghai, China) supplemented with 10% fetal bovine serum (FBS; Thermo Fisher Scientific, Shanghai, China) at 37 °C under 5% CO_2_. The CDV ZJ strain is a virulent strain that was isolated from dead or diseased minks by serial blind passages on Vero cells until obvious cytopathic effects (CPE) were produced (six rounds). The virus was propagated in Vero cells in DMEM supplemented with 2% FBS and used for cDNA cloning of the full-length cDNA.

### Plasmid construction

Total RNA was extracted from CDV-infected cells using an RNA extraction kit (Sangon Biotech Co., Ltd., Shanghai, China) according to the manufacturer’s instructions. First-strand cDNA synthesis was carried out with a ProtoScript® First Strand cDNA Synthesis Kit (New England Biolabs, Beijing, China) at 42 °C for 1 h. All primers for target gene amplification used in this study are listed in Table 1.

**Table 1:** Primer sequences for plasmids construction and identification of recombinant virus

Name	Sequence (5′ → 3′)	Orientation	Position
F1	GGAAAGGAATTCCTATAGTCACCAGACAAAGTTGGCTATGGATAG	+	1–25
R1	CAGACTCAGCCTCATTTGAGGTCCT	-	2686–2710
F2	CTCAAATGAGGCTGAGTCTGACAGT	+	2691–2715
R2	AACCAGGTGCACTGAGAGCCTGAGTTG	-	5264–5290
F3	GGCTCTCAGTGCACCTGGTTAGTCCTG	+	5371 − 5297
R3	ATAGTACATACCTTGGCTTTGGAATTC	-	7906–7932
F4	AAAGCCAAGGTATGTACTATAGCAGTGG	+	7913–7940
R4	GCACTTACGGTTTCATAGATCTCTATA	-	10,975–11,002
F5	ATCTATGAAACCGTAAGTGCATTTATA	+	10,983–11,009
R5	CTTTCCAGAAGGTCGGTGATAATGAAT	-	13,524–13,550
F6	ATCACCGACCTTCTGGAAAGTACCAAA	+	13,531–13,557
R6	TGGAGATGCCATGCCGACCCACCAGACAAAGCTGGGTATGATAAC	-	15,666–15,690
NF	TAAGCAGAGCTCGGGTACCAGATCTAGGGTCAATGATCCTACCTTAGA	+	63–78
NR	TCTGGATCCCCGCGGCCGCAGATCTGTTTGTTGGACCCGGGTCCTAA	-	1740–1760
PF	TAAGCAGAGCTCGGCTAGCCTCGAGCTTAGGACCCGGGTCCAACAAAC	+	1745–1761
PR	TGCTGGAATTCGGCTTGGGCTCGAGGAGAGGACTTAGGCTCTTGTGT	-	3400–3421
LF:	ATCATTTTGGCAAAGAATTCCTCGAGGGTACCCCCGGGGCGGCCGCAAATGGACTCTGTGTCAGTGAACCAGA	+	9030–9054
LR:	AGGGAAAAAGATCTGCTAGCTAATTAAGAGCTCTTTTTTTTCGTATAACCAAGTTTGATAGC	-	15,621–15,645
PMF	GACGATCATGCGATTGTTTA	+	8666–8685
PMR	TGTCTAATTCGTGCATACTC	-	9117–9136

The vector backbone, puCMV containing the human cytomegalovirus (hCMV) immediate early promoter and SV40 polyA signal, was constructed by our lab. puCMV was partially derived from pUC57, while some of sequences are artificially modified and commercially synthesized by GenScript. In terms of viral gene construction, puCMV was digested with the restriction enzymes PacI and NotI for CDV full genome insertion. The full-length cDNA clone puCMVZJ was generated with six overlapping fragments by the seamless cloning method. Briefly, primer pairs F1-F6 (Table 1) were used to amplify the target genes. Each DNA fragment shares a 20 bp terminal homology with the adjacent fragment for seamless cloning, and the plasmid puCMVZJ was made by homologous recombination using the GeneArt Gibson Assembly HiFi Cloning Kit (Thermo Fisher Scientific, Shanghai, China).

**Fig. 1:**
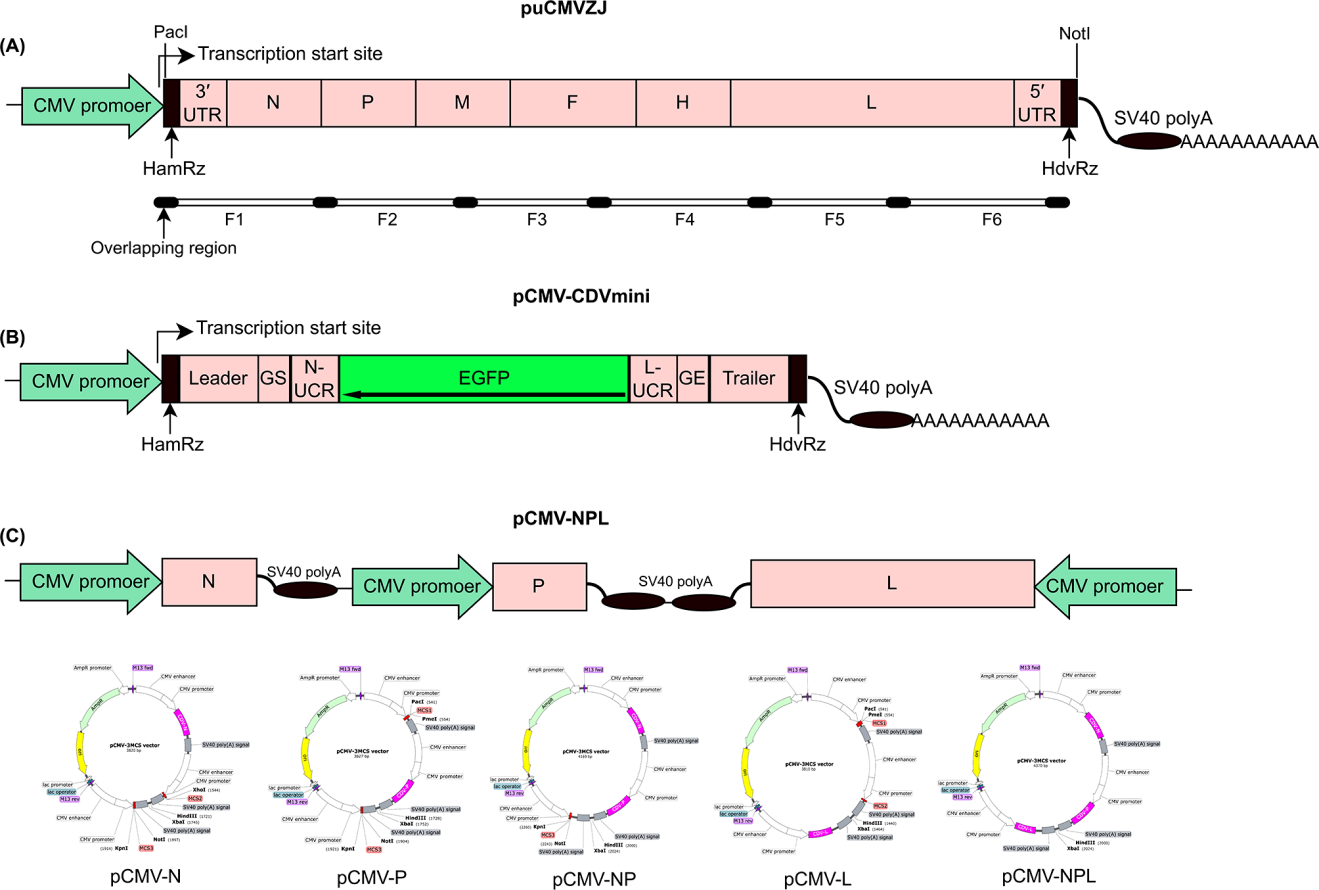
Schematic diagrams of the constructs used in the study. **(A)** The full-length cDNA clone of CDV ZJ. The full-length viral cDNA was flanked by hammer-head ribozyme (HamRz) and hepatitis delta ribozyme (HdvRz) sequences at both terminals of the viral genome. The six overlapping fragments and the overlapping regions are shown below the genome. Transcription of the plasmid is under the control of the CMV promoter and SV40 polyA signal. **(B)** Strategy for constructing the CDV minigenome. The minigenome is composed of the 3’ leader, the N gene start signals (GS), the noncoding region (NCR) of the N gene, EGFP, the 5′ NCR of the L gene, the L gene end signals (GE) and the 5′ trailer, which was inserted into the same vector used for the generation of vial cDNA clones. **(C)** Co-expression of CDV N, P, and L genes in one plasmid. The plasmid pCMV-3MCS was derived from the pUC57 vector by introducing three CMV promoters and polyadenylation signals, which contained three independent expression cassettes for multiple gene expression. Cloning strategies and individual plasmids (pCMV-N, pCMV-P, pCMV-NP, pCMV-L, and pCMV-NPL) are shown

To produce the exact 3’ and 5’ ends of the antigenomic RNA transcript, two self-cleaving ribozymes, hammerhead ribozyme (HamRz, TGTTAAGCGTCTGATGAGTCCGTGAGGACGAAACTATAGGAAAGGAATTCCTATAGTC) and hepatitis delta ribozyme (HdvRz, GGGTCGGCATGGCATCTCCACCTCCTCGCGGTCCGACCTGGGCATCCGAAGGAGGACGCACGTCCACTCGGATGGCTAAGGGAGGGCG), were inserted between the PacI and NotI sites and assembled with the 3’ UTR and 5’ UTR based on overlapping extension PCR (Fig. 1A). The N, P, and L genes were amplified and inserted into the multiple cloning sites in the pCMV-3MCS vector, which was derived from the plasmid pUC57 by inserting three CMV promoters and polyadenylation signals, resulting in plasmids pCMV-N, pCMV-P, pCMV-NP, pCMV-L, and pCMV-NPL (Fig. 1C).

The expression orientations of each gene are shown in Fig. 1C. To verify the function of pCMV-NPL and generate a negative-sense minireplicon RNA after transcription, a minigenome with EGFP in the antisense orientation was constructed by replacing all the viral encoding region with EGFP, designated pCMV-CDVmini (Fig. 1B). All plasmids were sequenced to verify their construction.

### Transfection

The day before transfection, BSR or Vero cells were seeded in 6-well plates at a density of 2 × 10^5^ per well. When the Vero cells were almost 80–90% confluent, the medium was replaced with fresh medium, and the cells were transfected with 2 µg of puCMVZJ and 3 µg of pCMV-NPL by using Lipofectamine 3000 transfection reagent (Thermo Fisher Scientific, Shanghai, China) according to the manufacturer’s instructions. At 24 h post-transfection, the cells were washed once and maintained in DMEM supplemented with 2% FBS for an additional 3–6 days until an obvious cytopathic effect (CPE) was observed. To examine the expression efficiency of pCMV-NPL, BSR-T7 cells were cotransfected with pCMV-NPL and pCMV-CDVmini at an appropriate ratio (1:1) and then incubated for 48–72 h at 37 °C with 5% CO2. The cells were examined daily by fluorescence microscopy.

### Identification and titration of rescued virus

The genomic RNA of rescued virus was isolated at the 10th passage (in Vero cells using DMEM with 2% FBS) with an RNA extraction kit and then subjected to RT-PCR (cDNA was generated by using ProtoScript II First Strand cDNA Synthesis Kit; New England Biolab, Beijing, China) analysis with primers PMF/PMR. The amplified fragment was purified for sequencing analysis. Positive recombinant virus should contain a restriction enzyme Pme I (nucleotides 8975–8982) in this amplified region, which was generated by introducing point mutations into the parental virus genome.

To determine virus yield, Vero cells were initially seeded at a density of 2 × 10^5^ cells/well in 96-well plates, and serially diluted viruses were added to 96-well plates and maintained at 37 °C for 4–7 days. The viral titers were measured by 50% end-point dilution (TCID_50_) assays according to the Reed-Muench method [15].

## Results

### The rescue efficiency of the established reverse genetic system

**Fig. 2:**
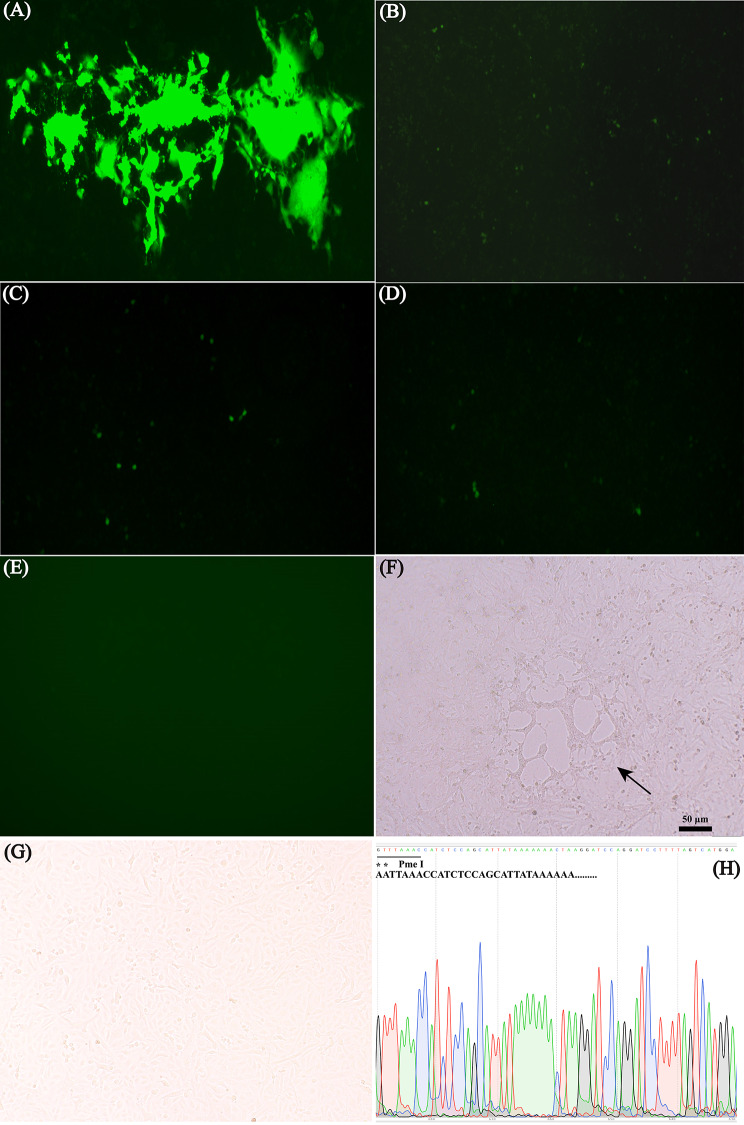
Analysis of plasmids transfection (magnification 100 x). **(A)** The minireplicon was produced by transfection of the pCMV-CDVmini plasmid into Vero cells, and RdRP was generated by virus incubation before plasmid transfection. The production and replication of viral microreplicons depend on the viral polymerase supplied by CDV-infected cells. **(B)**, **(C)**, **(D)**, and **(E)** Analysis of different rescue systems based on minigenome expression. (B) BSR cells were transfected with 2.0 µg of pCMV-CDVmini plasmid and 3 µg of pCMV-NPL plasmid. **(C)** BSR cells were transfected with 2.0 µg of pCMV-CDVmini plasmid, 1 µg of pCMV-NP and 1 µg of pCMV-L. **(D)** BSR cells were transfected with 2.0 µg of pCMV-CDVmini plasmid, 0.5 µg of pCMV-N, 0.5 µg of pCMV-P, and 1 µg of pCMV-L. **(E)** BSR cells were transfected with 2.0 µg of pCMV-CDVmini plasmid. **(F)** Cytopathic effect (CPE) induced in Vero cells co-transfected with plasmids pCMV-NPL and puCMVZJ. Arrows indicate syncytia in the cells that were observed at 5 days after transfection. The arrow indicates the formation of syncytia in the cells, which was observed 5 days after transfection. **(G)** Control cells. **(H)** Sequencing results of the mutation sites in the recombinant virus genome. Asterisks demonstrate that the A → G and A → T substitutions at viral nucleotides 8975 and 8976 to create a Pme I restriction site in the recombinant viral genome

To confirm whether pCMV-CDVmini was functional by its ability to transcribe CDV anti-minigenome RNA, Vero cells were initially infected with CDV at an MOI of 2. After 2 h of incubation, pCMV-CDVmini was transfected into Vero cells. At 72 h post-transfection, green fluorescence could be observed in CDV-infected cells transfected with pCMV-CDVmini (Fig. 2A). The results demonstrated that the plasmid was functional and could provide a cis-acting signal for viral gene transcription. Then, pCMV-CDVmini was used to co-transfect the BSR cells with pCMV-NPL to determine the expression of the N, P, and L genes. After 72 h, EGFP expression was detected in transfected cells (Fig. 2B), suggesting that pCMV-NPL has virus-like functions in viral genome transcription and can reconstitute a functional viral polymerase complex that acts on the minigenome. Furthermore, to test the efficiency of the established system, different rescue systems were evaluated based on minigenome expression, including a four-plasmid system and a three-plasmid system. As shown in Fig. 2C and E, compared with that of the other plasmid systems, more fluorescent cells were observed in the two-plasmid reverse genetic system at 72 h post-transfection (Fig. 2B). The results indicated that the transfection system with two plasmids had advantages such as ease of use, high transfection efficiency, and improved rescue efficiency of CDV.

### Characterization of rescued virus

**Fig. 3:**
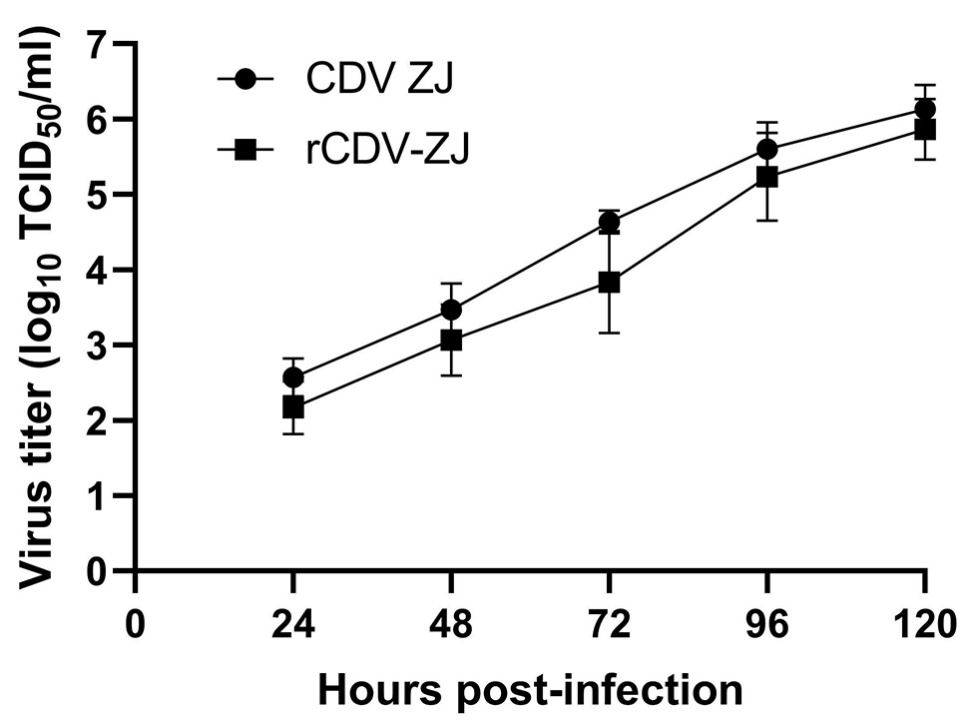
Growth curve comparison of CDV ZJ and rCDV-ZJ in Vero cells. Cells were infected with parental and recombinant virus at an MOI of 0.01 for 120 h. Viral titers were determined as the number of TCID_50_/ml in an endpoint titration assay. The data are shown as the mean of triplicates ± SE

To further test the rescue efficiency of the two-plasmid system, Vero cells were co-transfected with puCMVZJ and pCMV-NPL. Obvious syncytia were observed at 5 days after transfection (Fig. 2F), no cytopathic effect (CPE) was observed in negative control cells (Fig. 2G). The rescued viruses were continuously cultivated in Vero cells for 10 passages and were confirmed by RT-PCR. As expected, the sequencing results confirmed the presence of Pme I in the recombinant CDV (Fig. 2H). With the increase of infected passages, the titer of rescued virus also increased and was similar to that of the parent virus by the 7th passage (Fig. 3).

## Discussion

As a member of the genus *Morbillivirus*, CDV has similar characteristics to other viruses within the same genus, especially measles virus. They are all negative-strand RNA viruses, and the RNA genome is not infectious by itself. Therefore, the RNA genome cannot be transcribed into positive-strand RNA and is translated into proteins directly due to the lack of RdRP in cells. To overcome these obstacles and establish a rescue system in vitro, measles virus (MV) was first rescued in 1995 by developing a helper cell line (293-3-46) that stably co-expressed MV-N, MV-P, and T7 polymerase [16]. Subsequently, a modified vaccinia virus Ankara (MVA) that expresses T7 RNA polymerase was applied to rescue MVs based on a four-plasmid rescue system [17]. This approach requires the incubation of MVA-T7 before the plasmid transfection step for RNA transcription and protein expression. The drawbacks of this approach are the cytopathogenic effect and release of progeny vaccinia virus during virus rescue [18]. The reverse genetic strategies of CDV are the same as those of MV, and there are currently several ways to generate recombinant CDV [12, 19–21]. All of these rescue systems are achieved based on the four-plasmid cotransfection method, as well as the needs of helper cells, plasmids, or helper viruses that supply T7 RNA polymerase for transcription.

During the virus rescue process, if a cell that co-expresses N, P, and L is called an effective transfection cell, only when the plasmid containing the full-length genome of CDV is also transfected into this cell can the virus be rescued successfully. Therefore, the chance of four plasmids being transfected into one cell at the same time is much lower than that of two plasmids or one plasmid system. The one plasmid system has already succeeded in recovering several negative strand RNA viruses, such as Newcastle Disease virus and influenza A virus [22, 23]. Although the one-plasmid rescue system improved rescue efficiency, it requires suitable vectors that can accommodate fairly large gene inserts. In addition, the single-plasmid rescue systems may affect viral replication [22]. Based on this consideration, a two-plasmid rescue system was designed in this study by co-expressing the N, P, and L genes in a single plasmid. This approach has been previously reported for rescue of recombinant Newcastle disease virus and measles virus by co-expression of all helper proteins in a single-plasmid [24, 25]. Compared with the four-plasmid system and one-plasmid system, the transfection procedures, such as the ratio of plasmids and transfection reagent, of the two-plasmid system were easier to manipulate, and the transfection and rescue efficiency was relatively higher. In addition, the two-plasmid system can improve the production of rescued viruses and also be applied to rescue viruses that cannot be rescued by the 4-plasmid system [25]. In the current study, to eliminate the gene size effect of different expression cassettes on the expression level of the downstream proteins in the multigene expression system, a counterdirectional cassette was designed in the vector by adjusting the expression direction of the promoter.

## Conclusions

Reverse genetics is a powerful tool to produce modified viruses that have been successfully employed in vaccine development, gene therapy, drug screening, and other basic research [26–30]. Although CRISPR gene-editing technology can also be applied to manipulate and modify viruses, reverse genetic systems still have irreplaceable advantages in some aspects today, such as the insertion and replacement of long fragments, multisite knockouts, or mutations. In this study, we established a CDV rescue system by co-transfection of two plasmids, which is more convenient to use, easy to control, and of high rescue efficiency compared with the traditional reverse genetics system. This work provides a new strategy for improving the rescue efficiency of CDV and has the potential to be used in other morbilliviruses for viral rescue.

## Data Availability

All data generated and/or analyzed during this study are included in this manuscript. The raw data are available from the corresponding author upon reasonable request.
