# Correction: An improved system to generate recombinant canine distemper virus

**DOI:** 10.1186/s12917-024-04163-z

**Published:** 2024-07-03

**Authors:** Huai Cheng, Hewei Zhang, Huayun Zhang, Huanchang Cai, Min Liu, Mingen Yu, Meihua Xiang, Shubo Wen, Jingqiang Ren

**Affiliations:** 1 Wenzhou Key Laboratory for Virology and Immunology, Institute of Virology, Wenzhou University, Wenzhou, China; 2 College of Food and Drugs, Luoyang Polytechnic, Luo Yang, China; 3 Animal Diseases and Public Health Engineering Research Center of Henan Province, Luoyang, China; 4 Research and Development Department, Hangzhou Goodhere Biotechnology Co., Ltd, Hangzhou, China; 5 Preventive Veterinary Laboratory, College of Animal Science and Technology, Inner Mongolia Minzu University, Tongliao, China

**Correction:** ***BMC Vet Res*** **20, 162 (2024)**

10.1186/s12917-023-03830-x

Following the publication of the original article [1], it was noted that due to a typesetting error, the co-corresponding author name “Jingqiang Ren”, as well as the author’s email address “rjq207@163.com”, was not listed under the correspondence information field. The original article has been corrected.
